# Biosynthetic gas vesicles as a novel ultrasound contrast agent for diagnosing and treating myocardial infarction

**DOI:** 10.7150/thno.118543

**Published:** 2025-07-28

**Authors:** Zihang Wang, Maierhaba Yibulayin, Kezhi Yu, Tingting Liu, Lina Guan, Baihetiya Tayier, Lingjie Yang, Shangke Chen, Yuming Mu, Fei Yan

**Affiliations:** 1 Department of Echocardiography, The First Affiliated Hospital of Xinjiang Medical University, Xinjiang Key Laboratory of Ultrasound Medicine, Xinjiang 830000, China.; 2 Department of Ultrasound, Fifth Affiliated Hospital of Sun Yat-sen University, Zhuhai 519000, Guangdong, China; 3 Department of Ultrasound, The Second People's Hospital of Shenzhen, The First Affiliated Hospital of Shenzhen University, Shenzhen, P. R. China; 4 Xinjiang Key Laboratory of Ultrasound Medicine. No. 137, Liyushan South Road, Xinjiang 830000, China; 5 State Key Laboratory of Quantitative Synthetic Biology, Shenzhen Institutes of Advanced Technology, Chinese Academy of Sciences, Shenzhen 518055, China.

**Keywords:** myocardial contrast ultrasound, gas vesicles, ultrasound contrast agents, nanobubbles, myocardial infarction

## Abstract

**Rationale:** Myocardial contrast echocardiography (MCE) plays an important role in diagnosis of myocardial infarction (MI). However, its accuracy is limited by image quality because microbubble-based MCE produces negative contrast enhancement in the infarcted myocardial tissue. This study aimed to develop nanoscale gas vesicles (GVs) from *Halobacteria NRC-1* (hGVs) and GV-expressing genetically engineered *E. coli* (eGVs) and compare their imaging performance with commercial Sonovue in MI rats.

**Methods:** We developed nanoscale gas vesicles (GVs) from *Halobacteria NRC-1* (hGVs) and GV-expressing genetically engineered *E. coli* (eGVs) and compared their imaging performance with Sonovue in MI rats. Unlike SF₆-filled Sonovue, GVs are air-filled protein nanobubbles with unique shapes. We used immunofluorescence and TEM to examine GVs' distribution in myocardial tissue and analyzed the mechanisms of their penetration into infarcted areas. Additionally, we evaluated the potential of oxygen delivery to ischemic myocardium using ultrasound-targeted bubble destruction.

**Results:** hGVs produced significantly positive contrast enhancement and could last for a longer time in the infarcted area. Immunofluorescence and TEM examination confirmed that hGVs penetrated out the blood vessels into the ischemic myocardium and eGVs primarily retained around endothelial cells, while Sonovue could not pass through the damaged vessels. Mechanistic analysis revealed that inflammatory cytokines results in leaky blood vessels, facilitating the penetration of nanoscale GVs into the infarcted myocardial tissue. Moreover, hGVs exhibited excellent imaging performance across different pathological stages, especially during the inflammatory phase. More importantly, oxygen delivery into the ischemic myocardium through ultrasound-targeted bubble destruction technology greatly promoted the functional recovery of the ischemic myocardium.

**Conclusions:** hGVs demonstrated superior imaging performance and penetration capabilities specifically at the myocardial infarction sites in rats.Their ability to provide positive contrast and deliver oxygen via ultrasound-targeted bubble destruction enables improved diagnosis and treatment of MI.

## Introduction

Myocardial infarction (MI), known as “heart attack,” is one of the life-threatening coronary-associated pathologies characterized by sudden cardiac death 1. Pathologically, MI happens when blood flow is suddenly blocked, stopping oxygen from reaching the heart muscle and causing permanent myocardial damage 2,3. It's a medical emergency that requires immediate attention to prevent severe complications or death 4. Therefore, prompt diagnosis and early intervention are essential for improving patient survival chances 5,6. Recently, there are many methods used for diagnosis of MI, including electrocardiography (ECG), serum isoenzymes, physical assessment, and other noninvasive imaging techniques, etc 7-9. Chest pain is the first symptom of MI, but in some patients with silent ischemia, the disease can only be diagnosed in retrospect 10. Electrocardiography is the simplest diagnostic modality, with typical ST-T changes or abnormal Q waves during myocardial ischemia and infarction 11. These changes are easily recognized but not sensitive. Serum isoenzymes such as creatine kinase are a sign of myocardial necrosis, but its increase is delayed for a few hours after coronary occlusion 12. Perfusion nuclear studies enable physicians to diagnose MI, yet it is time-consuming 13. By contrast, echocardiography is powerful diagnostic modality and suitable in the emergency room. Especially, it can diagnose patients with no prior history of coronary artery disease whose electrocardiograms proved nondiagnostic 14,15.

With the development and utilization of contrast agents, myocardial contrast echocardiography (MCE) has been developed as a safe and effective technique to assess myocardial microcirculation 16,17. After injecting microbubble contrast agents into coronary arteries or peripheral veins, the infarcted region appears as a low signal while normally perfused areas show high signal, enabling direct assessment of the degree and adequacy of microvascular perfusion, and determining the area at risk and efficacy of reperfusion therapies in patients with MI 18,19. Also, MCE is an ideal tool for correct triaging of patients to revascularization and for assessing prognostic implications in patients with acute MI 20. However, the MCE-based evaluation is occasionally difficult due to the presence of acoustic attenuation, short duration, and insufficient enhancement of bubble signals relative to tissue 21,22. The main reason may attribute to the relatively large particle size of microbubbles, limiting their effectiveness to pass through the vessels to the infarcted area. Therefore, it is desirable to develop nanobubbles which can deliver into the infarcted region and produce high contrast signals in MI regions.

**Figure 1:**
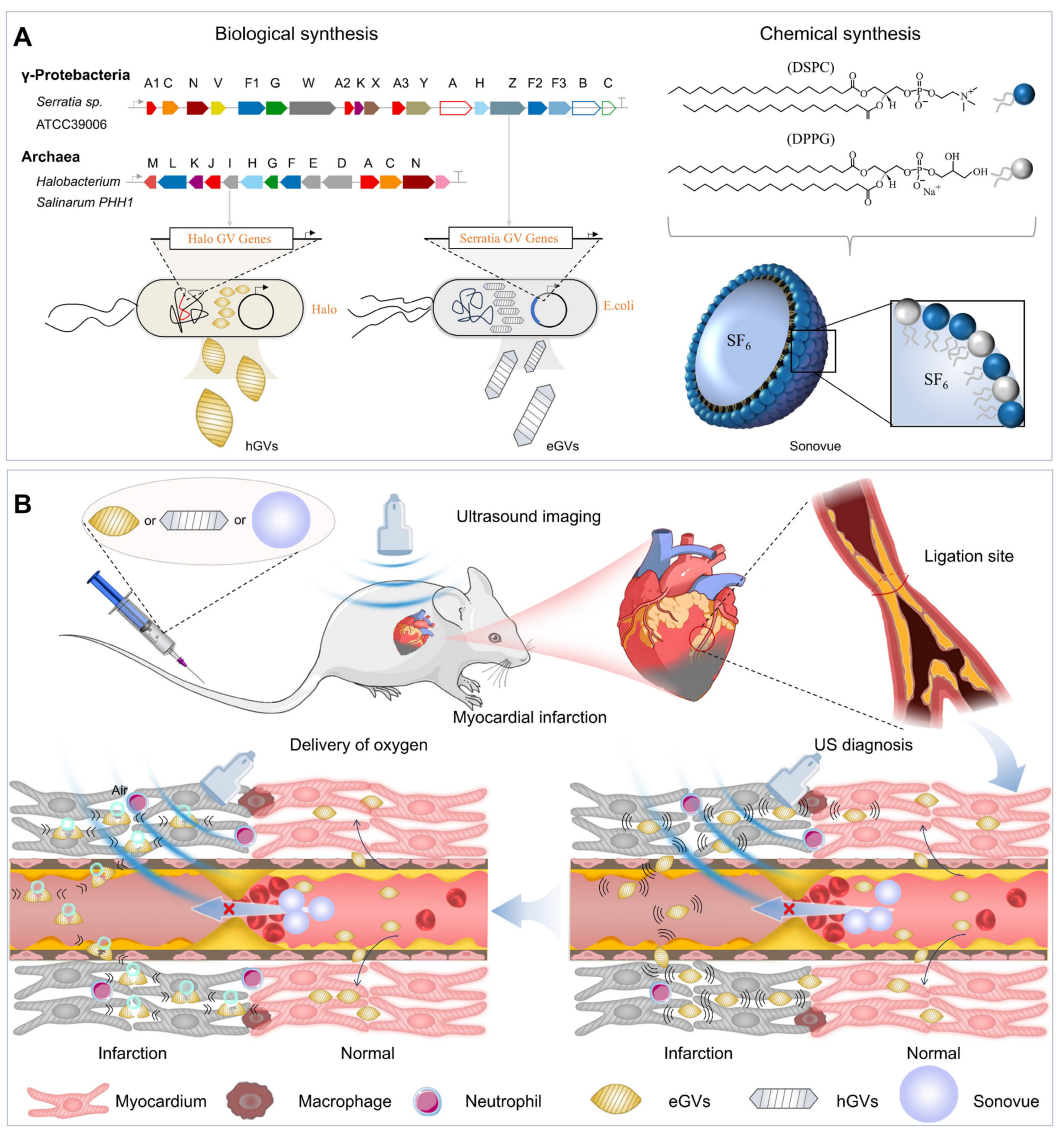
** Schematic illustration of biosynthetic GVs and commercially available Sonovue for their application in diagnosis and treatment of myocardial infarction.** (A) The different synthesis routes for hGVs, eGVs (biological synthesis) and Sonovue (chemical synthesis). The hGVs were extracted from *Halobacteria NRC-1* and eGVs were obtained from genetically engineered *E. coli* transformed with recombinant plasmids harboring gvp gene cluster from *Serratia 39006*, respectively. (B) Mechanism diagrams of the difference of biosynthetic GVs and Sonovue microbubbles in diagnosis and treatment of myocardial infarction. In the right panel, it can produce low or no acoustic signals in the infarcted area and high signals in surrounding normal myocardium after systemic administration of Sonovue microbubbles because the microsized particle size limits their availability to the infarcted myocardial tissue. In contrast, nanoscale hGVs and eGVs can infiltrate and accumulate into the ischemic myocardial tissue through extravasation because inflammatory responses after MI enlarge the gap between endothelial cells in the infarcted area. Importantly, the quick clearance of GVs from normal myocardial tissue produce apparent contrast signals in the infarcted area, making it possible to accurately diagnose MI. In the left panel, different from Sonovue with SF_6_ gas core, GVs have air core. The perfusion and accumulation of GVs in the ischemic myocardial tissue greatly facilitates oxygen gas delivery into the infarcted area when combining with ultrasound-mediated bubble destruction technology, providing additional oxygen supply to the ischemic myocardial tissue and helping their functional recovery.

Gas vesicles (GVs) from some planktonic microorganisms are promising alternatives to traditional acoustic contrast agents. They consist of a protein shell encoded by 10-14 Gvp genes, with an average particle size of about 100-200 nm 23. Our previous experiments showed that GVs produced stable and strong ultrasound contrast signals in mouse liver and tumors 24. Different with ball-shaped microbubbles, these GVs can reach ischemic tumor regions where traditional lipid microbubbles cannot penetrate after systemic administration 25. These results inspire us to explore the feasibility of GVs with different size and shape as nanoscale acoustic contrast agents for diagnosis of MI. To achieve this goal, we successfully synthesized two kinds of GVs with different particle sizes and shapes by biosynthetic methods. The one is hGVs derived from *Halobacteria NRC-1*, with spindle shape and approximately 200 nm particle size, and the other one is the heterologously expressed GVs (eGVs) from genetically engineered *E. coli* BL21 (A1) transformed with plasmids harboring the GVs-encoding gene cluster from *Serratia. 39006*, with cylinder shape and approximately 160 nm particle size 26,27. As the control, we used chemically synthesized Sonovue microbubbles, with ball shape and typical 2.5 μm particle size 28. Thanks to their small size, GVs can easily pass through the enlarged endothelial cell gaps and accumulate in the infarcted area where Sonovue cannot reach, producing high acoustic signals in the infarcted area because of accumulation of GVs and low or no signals in surrounding normal myocardium due to their quick clearance by blood circulation. More importantly, GVs carry oxygen-rich airs which can be delivered and released into the infarcted area when they receive high-power acoustic irradiation. This provides additional oxygen supply to the ischemic myocardium, greatly helping them to recover and to alleviate ventricular remodeling (Figure 1).

## Results and Discussion

**Figure 2:**
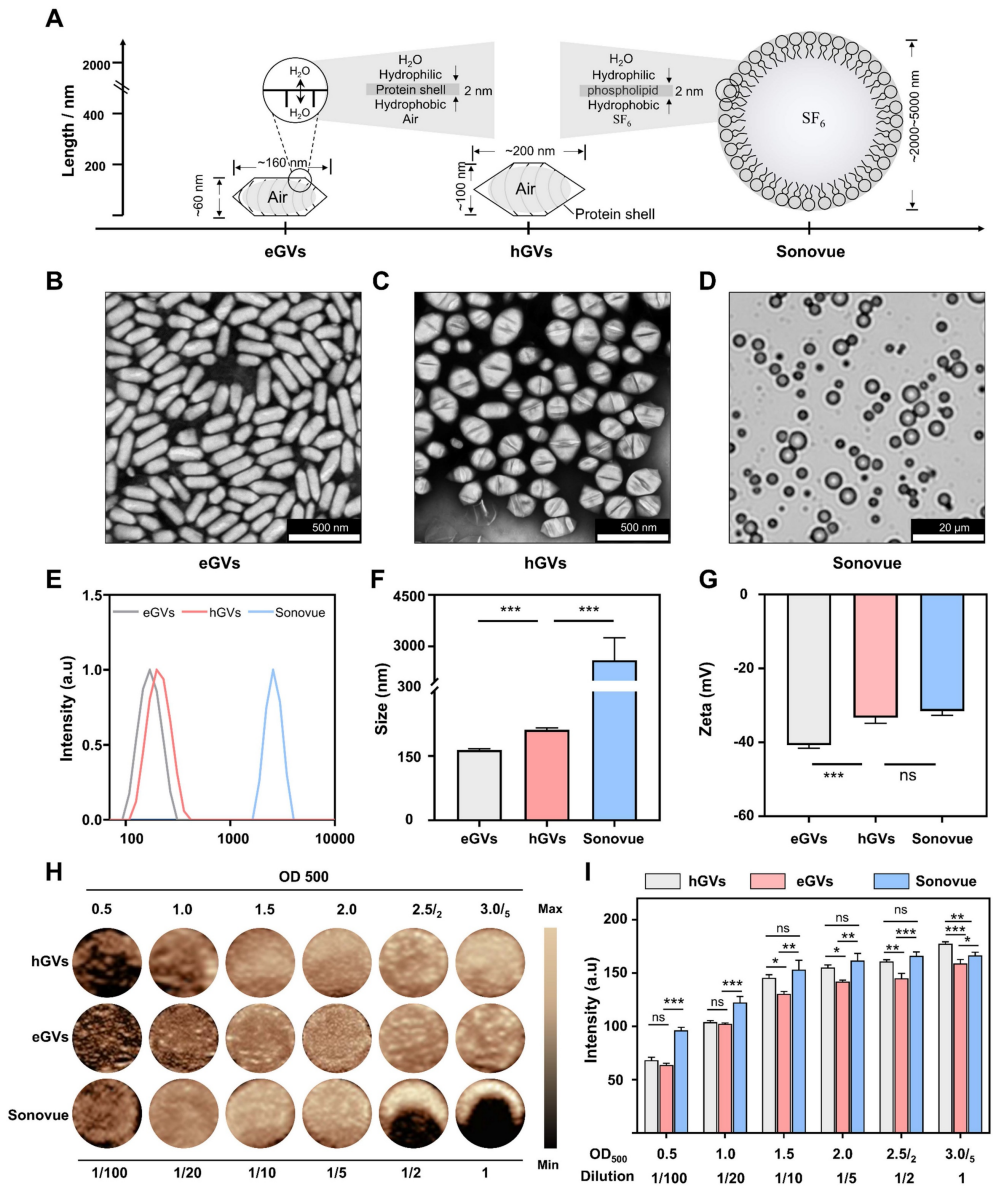
**Characterization of hGVs, eGVs and Sonovue.** (A) Schematic diagram illustrating the sizes and shapes of eGVs, hGVs and Sonovue. (B-C) TEM image of eGVs and hGVs. Scale bar: 500 nm. (D) Bright-field photograph of Sonovue. Scale bar: 20 µm. (E) Particle size distribution of eGVs, hGVs and Sonovue. (F-G) Particle sizes and zeta potentials of eGVs, hGVs and Sonovue. (n = 3). (H) In vitro imaging of eGVs, hGVs and Sonovue at various concentrations. (I) Quantification of in vitro imaging signal intensities of eGVs, hGVs and Sonovue at different concentrations. (n = 5). Data are presented as mean ± s.d. *P < 0.05, **P < 0.01, ***P < 0.001.

Characterization and comparison of GVs and Sonovue. Figure 2A shows the typical characteristics of biologically synthesized GVs and chemically synthesized Sonovue. Different from ball-shaped Sonovue with 2-5 μm particle size, eGVs are cylinder-shaped, with approximately 60 nm width and 160 nm length and hGVs are spindle-shaped, with about 100 nm width and 200 nm length (Figure 2B-D, Figure S1-S2). Also, both eGVs and hGVs have 2-nm protein shells made of gvpA and gvpC, with hydrophilic amino acids toward the outside aqueous phase and hydrophobic amino acids toward the inside gas phase. Furthermore, gas composition of GVs is different from Sonovue. The former including eGVs and hGVs contains air while the latter encapsulates SF_6_ inert gases. The GC-MS analysis of gas composition revealed approximately 27.30% O_2_, 70.29% N_2_ and 2.41% CO_2_ for hGVs and 25.59% O_2_, 71.06% N_2_ and 3.35% CO_2_ for eGVs, respectively (Figure S3-S4). Figure 2E-F showed the hydrodynamic diameter of these bubbles, revealing 163.9 ± 2.57 nm for eGVs, 208.3 ± 3.2 nm for hGVs, and 2600 ± 650 nm for Sonovue, respectively. Figure 2G shows that the zeta potential of Sonovue is -31.5 ± 0.9 mV, while the zeta potential of hGVs is -33.3 ± 0.24 mV, and that of eGVs is -40.7 ± 0.70 mV. To detect the imaging performance of GVs, the in vitro imaging of hGVs and eGVs was conducted at OD_500_ concentrations of 0.5, 1.0, 1.5, 2.0, 2.5/2 (two-fold dilution of GVs at OD_500_ = 2.5), and 3.0/5 (five-fold dilution of GVs at OD_500_ = 3.0). Additionally, in vitro imaging of Sonovue was performed at dilutions of 2-fold, 5-fold, 10-fold, 20-fold, and 100-fold. It can be clearly see that the imaging signal intensity of hGVs and eGVs gradually enhanced with the increase of the bubble concentrations similar with commercial Sonovue (Figure 2H). The quantitative results of in vitro imaging for hGVs, eGVs, and Sonovue at different concentrations are presented in Figure 2I, confirming the dose-dependent acoustic signals of hGVs and eGVs.

**Figure 3:**
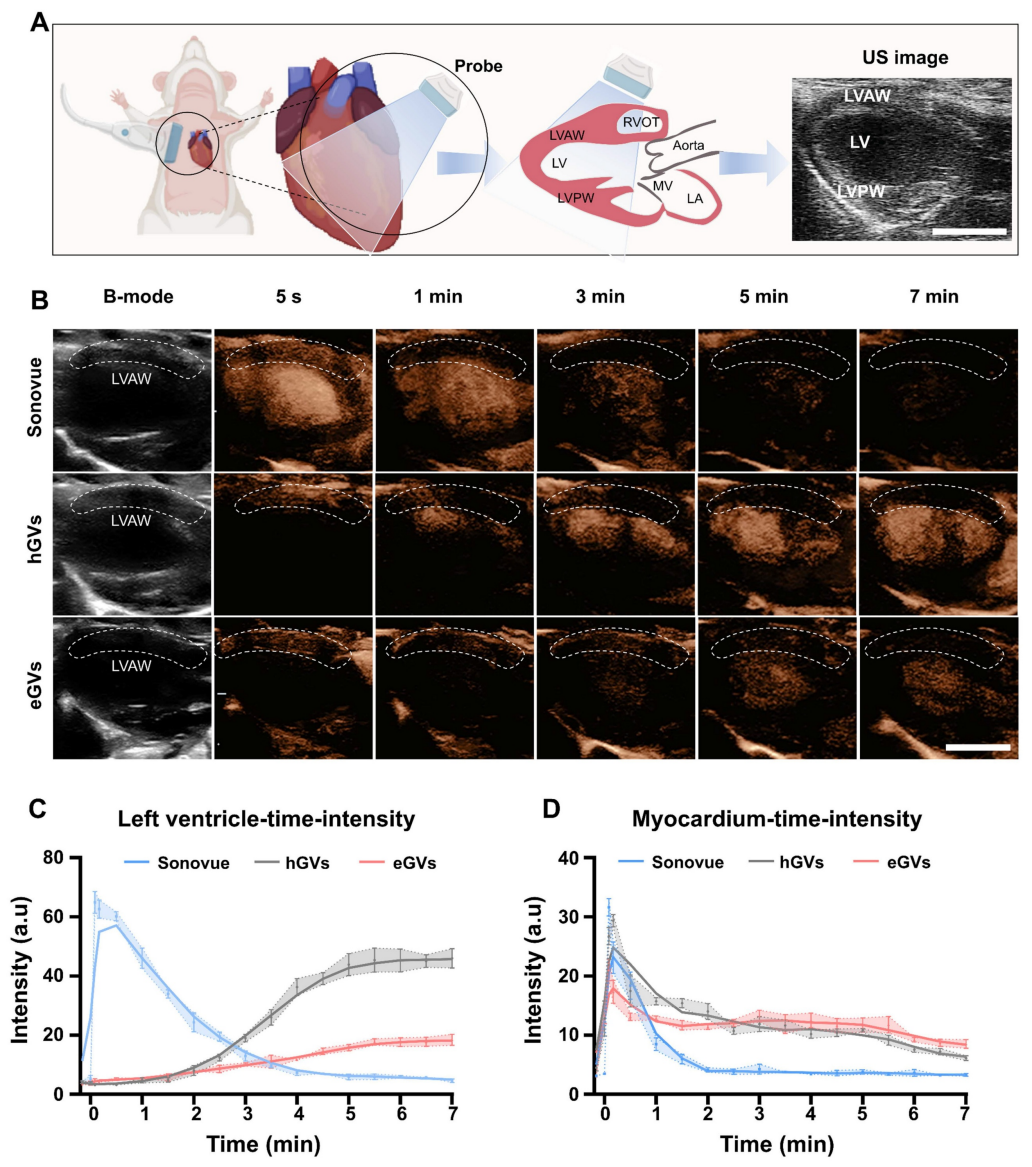
**Ultrasound contrast imaging of eGVs, hGVs and Sonovue in the normal rat hearts.** (A) Schematic diagram of the position of rat heart used for ultrasound imaging, showing the long-axis view of the left ventricle. Scale bar: 5 mm. It clearly depicts the anterior wall of the left ventricle, the cardiac cavity, and the posterior wall of the left ventricle. (B) Images of healthy rat hearts in B-mode and contrast mode after systemic injection of eGVs, hGVs or Sonovue at 5 s, 1 min, 3 min, 5 min and 7 min. Scale bar: 5 mm. (n = 3). (C) Time-intensity curves of the left ventricle demonstrate the dynamic changes in the contrast signals of the left ventricle after systemic injection of hGVs, eGVs or Sonovue. (n = 3). (D) Time-intensity curves of the anterior wall myocardium of the left ventricle after systemic injection of hGVs, eGVs or Sonovue. (n = 3).

**Myocardial imaging of GVs and Sonovue in healthy rats.** Subsequently, we used eGVs, hGVs and Sonovue to perform contrast-enhanced ultrasound imaging of healthy rat hearts. Figure 3A presented the schematic diagram of the normal cardiac cross section observed by the transducer, to better display the complete left ventricular anterior wall, left ventricle and left ventricular posterior wall. From Figure 3B, we can clearly see the location of the left ventricular anterior wall marked by white dotted boxes (the ROI) in the B mode. After intravenous administration of acoustic contrast agents, Sonovue could visualize the left ventricle and delineate endocardial border of the left ventricle within 5 s post-injection, reaching a peak and then gradually decayed within 5 min. Interestingly, hGVs first illuminated in the left ventricular anterior wall, and then gradually decayed within 3 min. Notably, no enhanced acoustic signals were observed in the left ventricle after immediate administration of hGVs. In contrast, the acoustic signals of the left ventricle gradually enhanced over time, reaching a peak after 7 min. Similar results were also observed in the heart of rats administered with eGVs. Figure 3C presents the quantitative acoustic signal intensity curves in the left ventricle received with Sonovue, hGVs or eGVs, revealing the peak intensity of 64.8 a.u at 5 s for Sonovue, 46.05 a.u at 7 min for hGVs and 18.44 a.u at 7 min for eGVs, respectively. Figure 3D showed the quantitative acoustic signal intensity curves in the left ventricle anterior wall, revealing the peak intensity of 31.62 a.u at 5 s for Sonovue, 29.35 a.u at 10 s for hGVs and 21.04 a.u at 5 s for eGVs, respectively (Supplementary Video 2,4,6). These data clearly indicated different acoustic contrast agents have different imaging and metabolic characteristics in the left ventricle and the left ventricular wall due to their different sizes and shapes.

**Figure 4:**
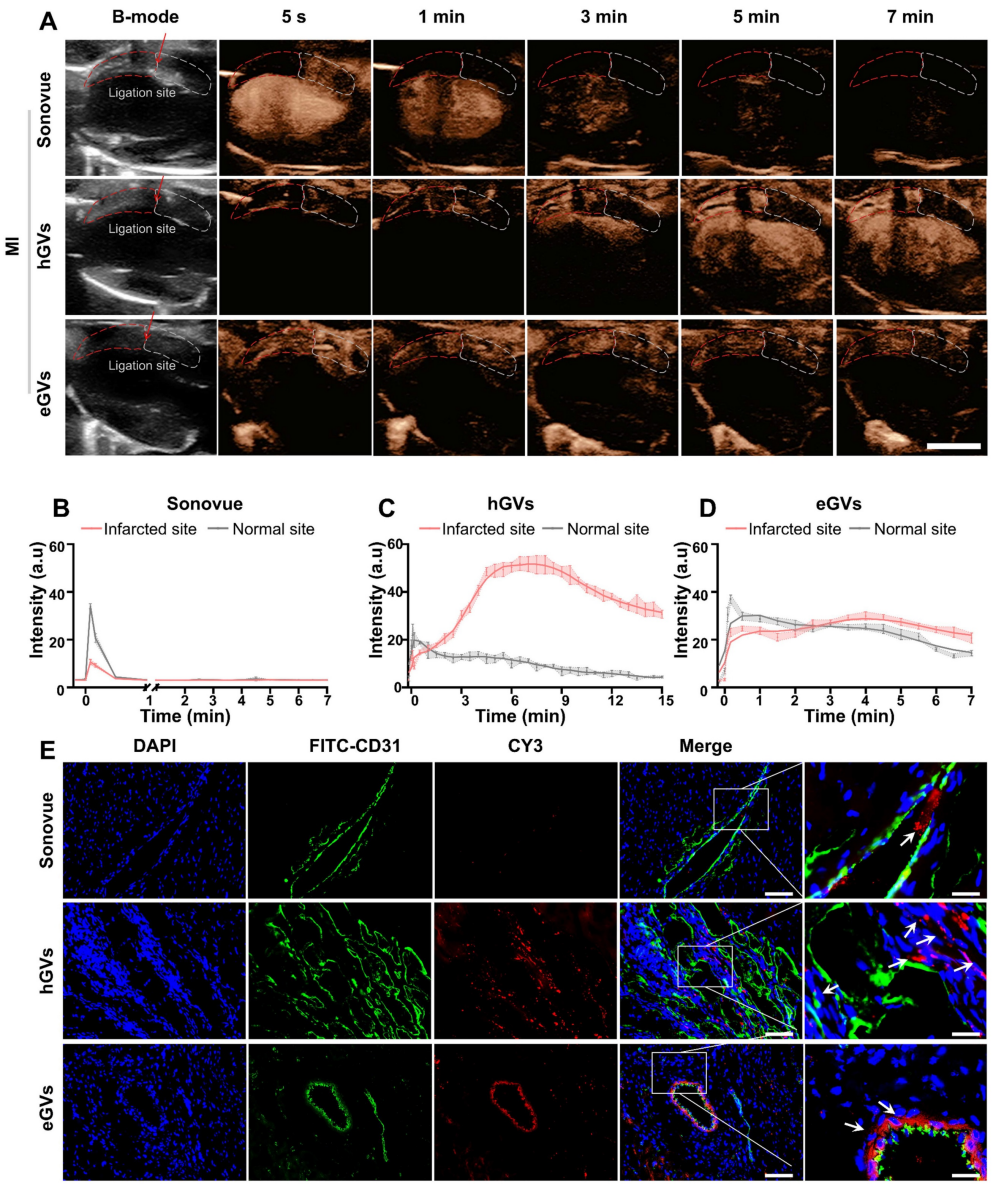
**Imaging and tissue distribution of hGVs, eGVs and Sonovue in rats with MI.** (A) Images of rat hearts with MI in B-mode and contrast mode after systemic injection of eGVs, hGVs or Sonovue at 5 s, 1 min, 3 min, 5 min and 7 min. Scale bar: 5 mm. (B-D) Quantitative analysis of contrast signal intensities in the infarcted area and the surrounding normal myocardial area within 7 min or 15 min after systemic injection of eGVs, hGVs or Sonovue. 7 min for eGVs and Sonovue and 15 min for hGVs. (n = 3). (E) Distribution of hGVs, eGVs and Sonovue in microvessels and myocardium within the infarcted area. Scale bar: 80 µm, Scale bar for enlarged image: 20 µm.

**Myocardial imaging of GVs and Sonovue in MI rats.** Next, we further compared the contrast-enhanced ultrasound imaging of eGVs, hGVs and Sonovue in rats with MI. For the convenience of observation, we positioned the transducer to facilitate to display the ligation site in the left ventricular anterior wall. From the Figure 4A, we can see that it is difficult to distinct infarcted myocardium from normal myocardium in the B-mode images. As expected, systemic administration of Sonovue could clearly show black myocardial infarct area (red dotted box) due to the absence of contrast signals relative to the non-infarcted normal myocardium (white dotted box). However, it is also worth noting that the difference of contrast signals between infarcted myocardium and normal myocardium only lasted for 1 min due to the rapid decline of Sonovue in normal myocardium. A huge decline of contrast signals also occurred in the left ventricle after 3 min post-administration until the contrast signal difference almost completely disappeared after 5 min. By contrast, systemic administration of hGVs exhibited a gradually increasing signal in the infarcted myocardial region of the left ventricular anterior wall, while the contrast signals in non-infarct areas tended to diminish over time. A gradually increasing signal could be observed in the left ventricle, achieving a peak after 7 min. Similar signal enhancement was observed in the left ventricular anterior wall after administration of eGVs, but the contrast signal of eGVs declines slower in the normal myocardium relative to hGVs. The obvious signal difference between the infarcted and normal myocardial tissues could be observed after 5 min post-administration. Notably, hardly signal enhancement was observed in the left ventricle after administration of eGVs.

Figure 4B-D showed the time-intensity curves of three contrast agents in the infarcted and normal myocardial tissues, revealing that Sonovue only displayed a short-time negative enhancement (30 s) in the infarcted myocardial tissue, with -23.51 a.u peak signal intensity difference relative to normal myocardial tissue (Figure 4B). In contrast, hGVs provided prolonged positive enhancement (15 min) in the infarcted myocardial tissue, with 40.53 and 26.29 a.u signal intensity differences relative to normal myocardial tissue after 7 min and 15 min, respectively (Figure 4C). Similarly, eGVs generated ultrasound contrast signals in myocardial tissue, with a duration of up to 7 min. However, the signal intensity difference between infarcted and normal myocardial tissue was minimal, with only +6.32 a.u. at 7 min (Figure 4D). Area under the curve (AUC) analysis of the time-intensity curves in the infarcted and non-infarcted tissues revealed significant differences for hGVs and Sonovue, but not for eGVs (Figure S5). In order to further confirm these acoustic signals of the infarcted myocardial tissues deriving from bubbles, we labeled hGVs, eGVs and Sonovue with red fluorescence dye and used for systemic administration. Tissue sections were collected 30 min after the injection of the contrast agent for immunofluorescence observation. Figure 4E demonstrated that hGVs could penetrate the damaged blood vessels and reached to the ischemic myocardial tissue while eGVs primarily accumulated around damaged endothelial cells, only with a small amount in the myocardial tissue. In contrast, Sonovue was confined in the blood vessels and could not penetrate into the surrounding myocardial tissue (Figure S6, Supplementary Video 1, 3, 5). These results indicate that the long-duration signal enhancement is attributable to the penetration and retention of hGVs into the infarcted myocardial tissue.

**Figure 5:**
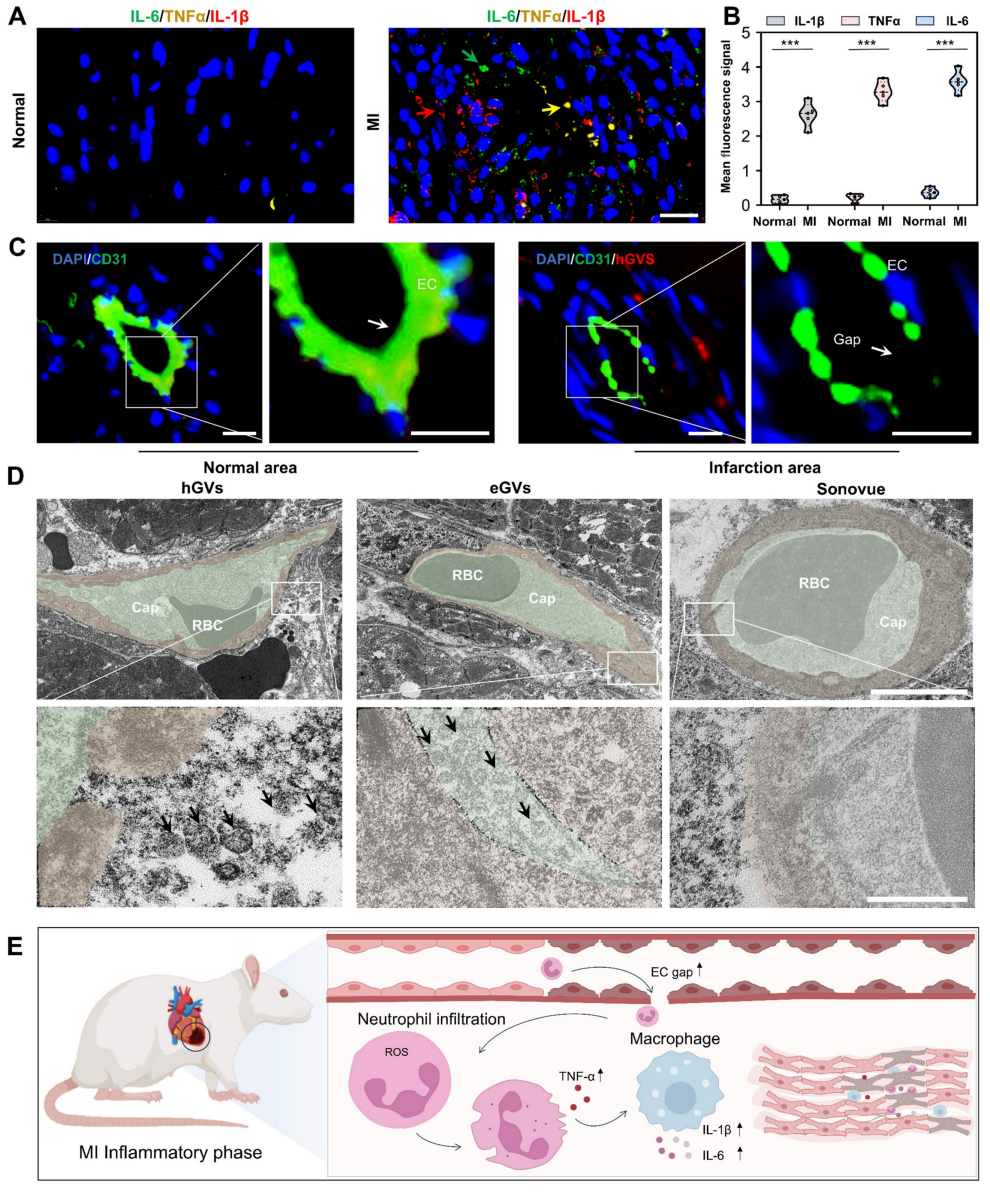
**Expression of pro-inflammatory cytokines and destruction of microvessels in the infarcted myocardial tissue**. (A) Immunofluorescence images of pro-inflammatory cytokines IL-6, TNF-α and IL-1β in the left ventricular anterior wall of the normal and MI rats. Scale bar: 20 µm. (B) Quantification of pro-inflammatory cytokines IL-6, TNF-α and IL-1β (n = 5). (C) Immunofluorescence staining of microvessel endothelial cells in the left anterior wall of the normal and MI rats. Green fluorescence indicates CD31-positive cells, while blue fluorescence indicates DAPI-stained nuclei. Scale bar: 10 µm. (D) Transmission electron microscopy images of microvessels at the infarcted myocardial tissue after injection of hGVs, eGVs or Sonovue. The bottom row of images represents a 10× magnification of the areas within the white boxes in the top row of images. Scale bars: 5 µm in the upper row and 500 nm in the bottom row, respectively. (E) Schematic diagram of mechanisms about the increased intercellular gap between endothelial cells and the accumulation of pro-inflammatory cytokines in the infarcted area after MI. Data are presented as mean ± s.d. *P < 0.05, **P < 0.01, ***P < 0.001.

**Mechanisms of GVs penetration into infarcted myocardial tissue.** To explore the reasons why hGVs can specifically enhance the contrast signals in the infarcted myocardial tissue but not in the normal myocardial tissue, we performed a pathological examination of the heart muscle at the myocardial infarction site. Significantly high-level expression of inflammatory cytokines including interleukin-6 (IL-6), tumor necrosis factor-alpha (TNF-α), and interleukin-1 beta (IL-1β) could be observed in the infarcted myocardial tissue, achieving 9.73- 15.09-, 15.71-fold higher than those of the normal myocardial tissue (Figure 5A-B). These results indicate that inflammatory responses occurred at the infarcted site. CD31 immunofluorescence staining analysis revealed that the integrity between vascular endothelial cells was damaged in the infarcted myocardial region, with numerous endothelial cells losing from the vascular endothelium, eventually leading to an enlarged gap between endothelial cells (Figure 5C and Figure S7). Transmission electron microscopy analysis further revealed apparent gaps existing in the round microvessels in the infarcted area. Notably, we found that hGVs can traverse these gaps to enter the ischemic myocardial tissue while eGVs mostly adhere to the endothelial sites. No Sonovue microbubbles were observed in the infarcted myocardial tissue, indicating that Sonovue could not cross the damaged vascular wall into the infarcted area (Figure 5D, Figure S15). Thus, upon the occurrence of MI, acute inflammatory responses would be induced, and a large number of neutrophils and other inflammatory cells such as macrophages would infiltrate into the ischemic myocardial tissues. These inflammatory cells secreted and produced numerous inflammatory cytokines, leading to blood vessel dilatation and the death of endothelial cells, eventually producing more permeable blood vessels which enhanced the penetration and retention of hGVs (Figure 5E). Evidences demonstrated that the elevated levels of TNF-α and IL-1β in the infarcted myocardial tissue may activate JAK-STAT and NF-κB signal pathways and increase the expression of adhesion molecules, favoring the binding of neutrophils and macrophages to the endothelium and increasing the vascular permeability 29.

**Figure 6:**
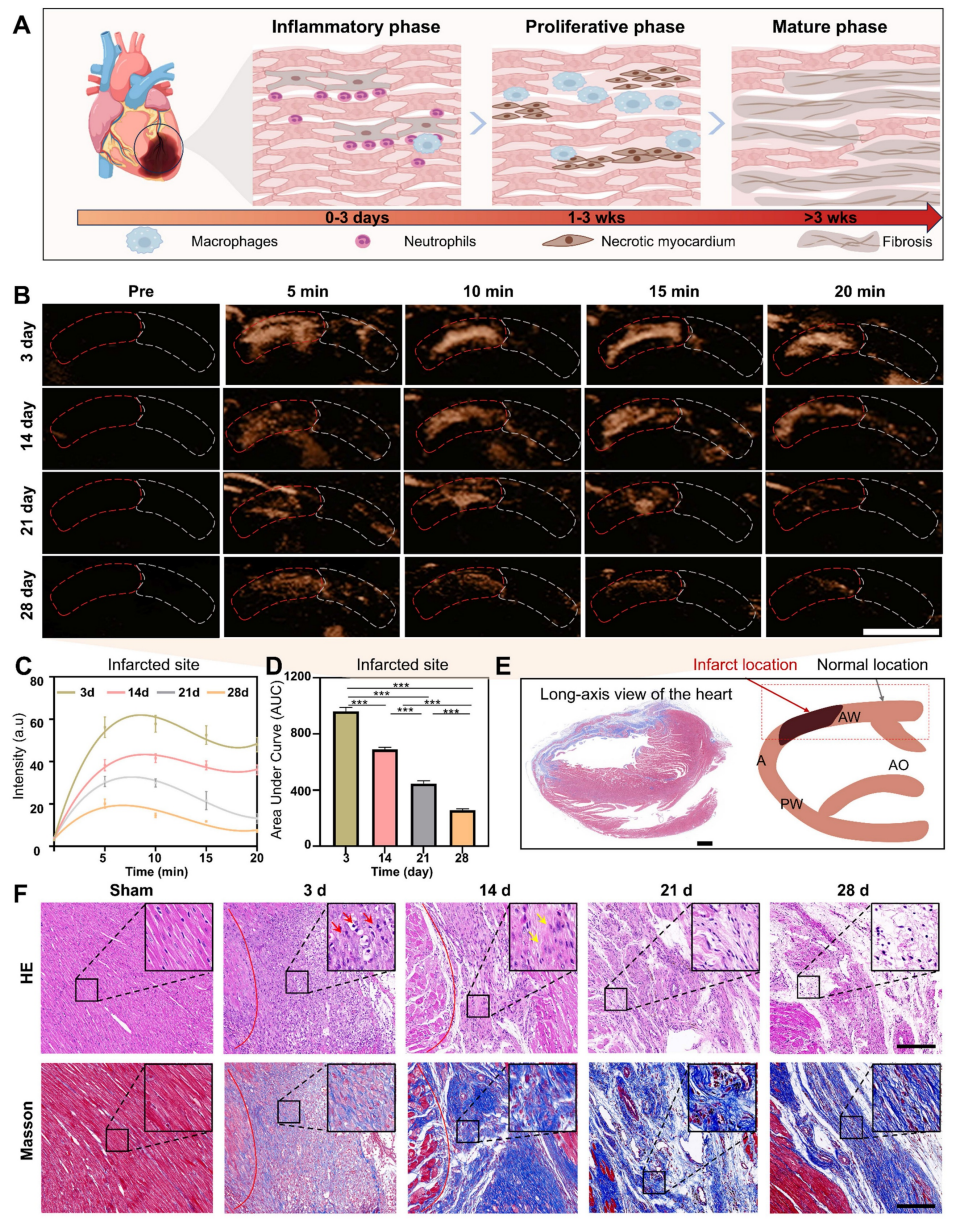
** Imaging of hGVs in the anterior wall of the left ventricle at different pathological stages after MI**. (A) Schematic diagram depicting the pathological progression at the infarct site. (B) The contrast-enhanced ultrasonography of left parasternal long-axis left ventricular wall after systemic injection of hGVs at 3, 14, 21, and 28 days post myocardial infarction in rats. Scale bar: 5 mm. (C) Quantitative contrast signal intensities of hGVs at the infarct site during various pathological stages. (n = 3). (D) Quantitative area under the curve (AUC) corresponding to the data shown in Figure c. (n = 3). (E) Masson's staining images of the left ventricular long-axis view and the schematic diagram corresponding to ultrasound section, with the black area indicating the infarct location. Scale bar: 1 mm. (F) H&E and Masson's trichrome staining of the myocardial infarction tissues from the Sham, 3-day, 14-day, 21-day, and 28-day groups. Scale bar: 200 µm. Data are presented as mean ± s.d. *P < 0.05, **P < 0.01, ***P < 0.001.

**Assessment of the degree of myocardial recovery.** The assessment of the degree of myocardial recovery is of utmost clinical importance since it is a predictor of prognosis for restoration of myocardial contractile function after revascularization. A typical progression of pathological changes during myocardial recovery over time after an infarction was schematically presented in Figure 6A, illustrating the transition through the inflammatory phase (creating inflammation as a defense response), proliferative phase (reparative cells help reduce inflammation), and maturation phase (healing through the formation of scar tissue). Considering that hGVs exhibit more excellent performance than eGVs in marking myocardial infarction, we selected hGVs for late-stage evaluation of myocardial recovery. To test the usability of hGVs in different myocardial recovery phases after an infarction, we systemically administrated hGVs into the rats after 3, 14, 21 and 28 days post-MI. Significantly enhanced acoustic signals could be observed in the infarcted area but not the non-infarcted area at all tested time points (Figure 6B, Figure S8, Supplementary Video 7). Quantitative analysis revealed the contrast signal intensities of the infarcted area changed over time after the systemic injection of hGVs, and all of them had peak signals about 5-10 min for different pathological stages (Figure 6C). Figure 6D presented the area under the curve (AUC) corresponding to four different pathological stages, demonstrating that there was a gradual decrease of contrast signal intensities in the infarcted area along with pathological progress (Figure 6D, *P < 0.001*). The long-axis view of the left ventricle showed the formation of fibrosis at the infarct site in the left ventricular anterior wall (indicated by the blue area), corresponding to the black area in the schematic diagram (Figure 6E).

To further elucidate the mechanisms underlying the declined contrast signal intensities of the infarcted area during the proliferative and maturation phases, we analyzed the pathological changes based on the histological staining by comparing the myocardial infarct areas of the sham-operated rats with those of rats at 3, 14, 21 and 28 days post-MI. Histological changes were clearly observed at these different time points, revealing significantly more inflammatory cells infiltrated in the myocardial infarct area after 3 days post-myocardial infarction (Figure 6F, inset in upper row). Patchy necrosis of myocardial cells, characterized by nuclear dissolution and eosinophilic cytoplasm, accompanied by a small number of infiltrating inflammatory cells, could be observed in the myocardial infarct area after 14 days post-MI. After 21-28 days, extensive patchy necrosis of myocardial cells was observed, with some areas undergoing dissolution and being replaced by proliferative connective tissue (Figure 6F, upper row). To further examine the distribution pattern of myocardial fibers and collagen fibers in the infarct region, we performed Masson trichrome staining analysis. Results showed that the degree of myocardial fibrosis gradually increased with the progression of pathology, with a small amount of blue collagen fibers surrounding the myocardial cells 3 days after MI. A large number of cardiomyocytes were replaced by fibrous tissue 14 days after MI. After 21-28 days, the cardiomyocytes cells were replaced by permanent collagen scars (Figure 6F, bottom row).

**Figure 7:**
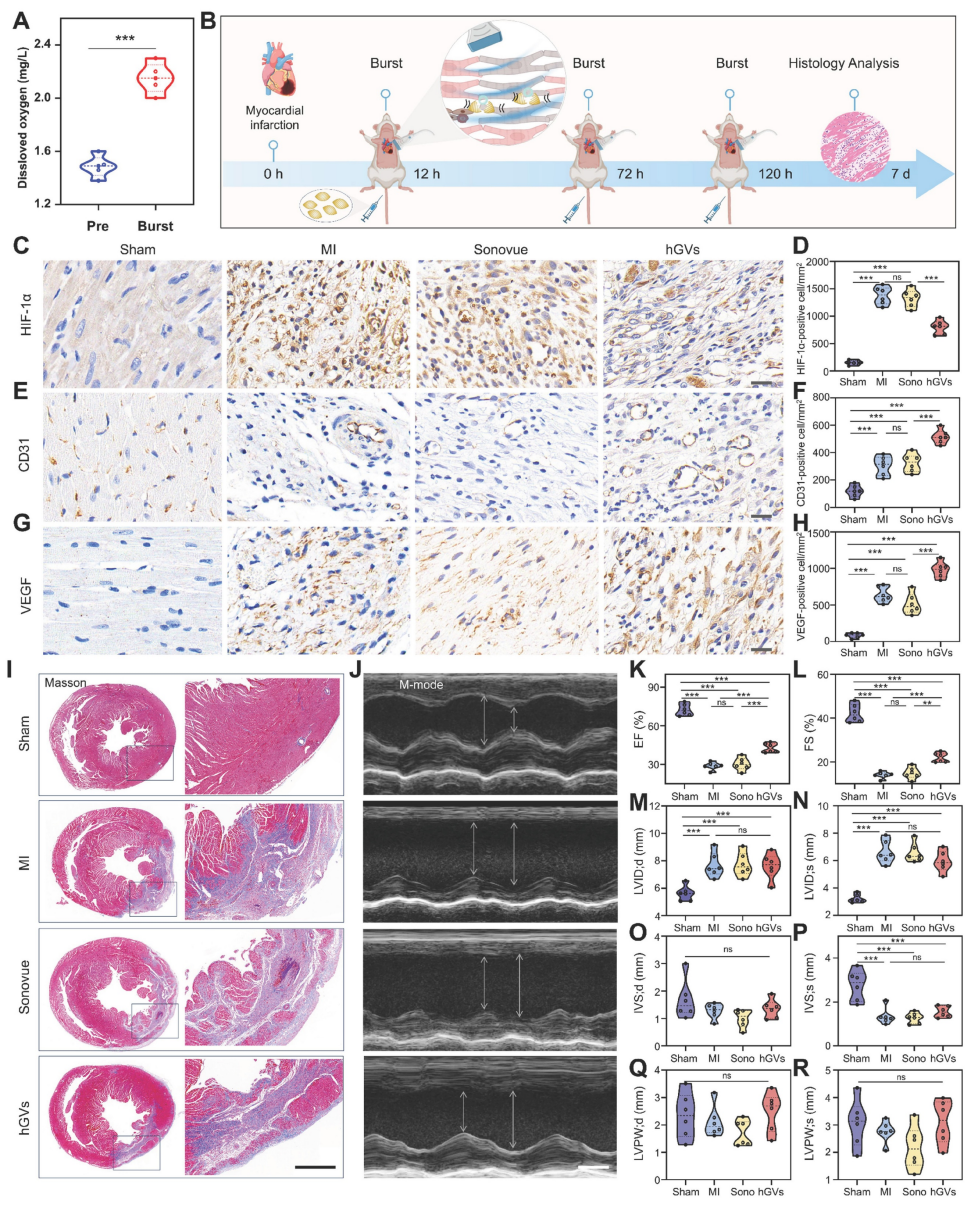
**Assessment of the therapeutic effect of oxygen delivery by hGVs in MI rats**. (A) The amount of oxygen in the solution of hGVs before and after acoustic irradiation. (B) Schematic diagram of therapeutic procedure delivering oxygen by hGVs combined with ultrasound irradiation. (C-H) Immunohistochemical staining and quantitative analysis of HIF-1α (C, D), CD31 (E, F) and VEGF (G, H) in the infarcted myocardial tissues from Sham, MI, Sonovue, and hGVs groups after treatment. Scale bar: 20 µm. (I) Masson's staining of Sham, MI, Sonovue, and hGVs groups after treatment. Scale bar: 1 mm. (J) Ultrasound M-mode imaging of Sham, MI, Sonovue, and hGVs groups after treatment. Scale bar: 3 mm. (K-R) Ejection fraction (EF) and fractional shortening (FS), left ventricular internal diameter at end-diastole (LVID; d) and end-systole (LVID;s), interventricular septum thickness at end-diastole (IVS;d) and end-systole (IVS;s), and left ventricular posterior wall thickness at end-diastole (LVPW; d) and end-systole (LVPW;s) in the Sham, MI, Sonovue, and hGVs groups after treatment. (Data are presented as mean ± s.d. n = 6 *P<0.05, **P<0.01, ***P<0.001).

**Oxygen delivery by hGVs promote functional recovery of infarcted hearts.** Considering the specific accumulation of hGVs in the infarcted myocardial tissues and the oxygen-rich gases in the hGVs, it inspired us to speculate that hGVs can be beneficial for the oxygen supply to ischemic myocardium through ultrasound-mediated targeted delivery technology, which would promote functional recovery after myocardial infarction. To verify this hypothesis, we firstly assessed the oxygen content in the solution of hGVs at OD500 = 2.0 before and after acoustic irradiation to destruct these hGVs. Significantly increased oxygen concentrations could be detected in the solution of hGVs after acoustic irradiation, achieving a 52.5% increase relative to non-irradiated hGVs (Figure 7A).

Next, we established the MI rat model and intravenously administrated hGVs or Sonovue after 12 h, 72 h and 120 h post myocardial infarction, followed by acoustic irradiation to the infarcted site under the guide of ultrasound imaging (Figure 7B). Immunohistochemical staining analysis of the infarcted myocardial tissues demonstrated that significantly higher levels of HIF-1α were observed in the untreated MI and Sonovue-treated groups in comparison to the Sham group, indicating these myocardium kept in ischemic state. Notably, the hGVs-treated group showed a significantly lower HIF-1α level compared to the MI group (Figure 7C-D). Higher level of CD31 expression was also observed in the hGVs-treated group, achieving 515 CD31-positive cells/mm^2^ vs 120 cells/mm^2^ for Sham group, 305 cells/mm^2^ for the MI group, and 325 cells/mm^2^ for Sonovue group (Figure 7E-F). Significantly more VEGF-positive cells (981 cells/mm^2^) were also found in the hGVs-treated ischemic myocardium vs 85 cells/mm^2^, 640 cells/mm^2^, 515 cells/mm^2^ VEGF-positive cells in Sham, MI, Sonovue groups, respectively (Figure 7G-H). Since CD31 is a marker of endothelial cells and VEGF functions as a key angiogenic factor, their upregulation indicates an enhanced endothelial function and potential neovascularization, which is critical for myocardial recovery. Moreover, we examined the morphology of mitochondria in the infarcted myocardial tissue. Significant mitochondria swelling and blurred cristae structures could be observed in the untreated control group and the Sonovue group. In contrast, normal mitochondria morphology with clear and well-aligned cristae could be seen in the hGVs plus acoustic irradiation group (Figure S16).

Masson staining of these heart tissues showed that the fibrotic area was significantly reduced in the hGVs group, compared with Sonovue and MI groups (Figure 7I, Figure S9). More importantly, we found that treatment with hGVs plus acoustic irradiation could significantly increase the ejection fraction (EF) and fractional shortening (FS) of MI rats (Figure 7K-L). Considering that the left ventricular dimensions and wall thickness function as indicators of myocardial remodeling. We measured these parameters at end-diastole (LVID;d, IVS;d, LVPW;d) and end-systole (LVID;s, IVS;s, LVPW;s), revealing no significant differences among MI model groups (Figure 7M-R). These data indicates that the acoustic interventions can recover the cardiac function but not significantly impact ventricular structure.

**Biosafety evaluation.** To evaluate the biosafety of hGVs, H&E histological staining analysis was performed for the heart, liver, spleen, lung and kidney of rats systemically administrated with hGVs or PBS, showing no obvious morphological damage to these organs in the hGVs-treated group (Figure S10). Additionally, serum biochemical tests on these hGVs-treated rats revealed that levels of ALT (alanine aminotransferase), AST (aspartate aminotransferase), urea (UREA), creatinine (CREA), and blood urea nitrogen (BUN) remained within normal ranges, indicating that the systemic administration of hGVs did not cause any damage to these rats (Figure S11).

## Conclusion

In this study, we comprehensively compared the physicochemical properties and imaging performance of biologically synthesized GVs and commercially available Sonovue which was chemically synthesized in the rat hearts. Through morphological and gas composition analysis, we found that GVs significantly differed from Sonovue in terms of particle size, shape and gas composition. Both eGVs and hGVs were air-filled protein nanobubbles with cylinder or spindle shape structure, while Sonovue was SF_6_-filled lipid microbubbles with spherical shape structure. When used for imaging of healthy rat hearts, Sonovue quickly delineated the left ventricle and its boundaries, but the signal enhancement duration only lasts for 1-2 min. In contrast, hGVs and eGVs show slower but longer signal enhancement duration in cardiac imaging. Further studies in MI rats demonstrated that Sonovue could only provide negative contrast enhancement in the infarct area for a short period (< 1 min), while hGVs and eGVs produced positive contrast enhancement and could last for 7 min. Notably, hGVs showed the longer signal duration and stronger enhancement in the infarcted area in comparison with eGVs, which may be attributed to the cylinder-shaped structure of eGVs and their lower vascular permeability, leading to reduced contrast imaging performance.

Immunofluorescence and TEM examination confirmed that hGVs penetrated out the damaged blood vessels and accumulated in ischemic myocardium, while eGVs primarily retained around endothelial cells, and Sonovue could not pass through the damaged vessels. Mechanistic analysis revealed high level inflammatory cytokines, including interleukin-6 (IL-6), tumor necrosis factor-alpha (TNF-α), and interleukin-1 beta (IL-1β), could be observed, resulting in the death of endothelial cells and the leaky blood vessels, and eventually enhancing the penetration and retention of hGVs and eGVs into the infarcted myocardial tissue. Also, we demonstrated hGVs exhibited excellent imaging performance across different pathological stages, especially during the inflammatory phase. Compared to conventional nanocarriers (e.g., liposomes, exosomes), hGVs offer distinct advantages, including superior biocompatibility, high gas-loading capacity, and the vasculature penetration ability. Thus, hGV-based strategies can provide a non-invasive, controllable, and clinically translatable approach for gas therapy.

## Materials and Methods

**Synthesis and extraction of eGVs and hGVs:** The GVs-encoding gene cluster was cloned from *Serratia 39006* and inserted into the pET28a (+) vector via Gibson assembly technique. The gene sequence was verified by Sanger sequencing (Figure S13), and the recombinant plasmid was transformed into *E. coli* BL21(A1) competent cells, followed by cultivation in LB medium with 50 μg/mL kanamycin at 37 °C for 16 h. Once the OD_600_ reached 0.6-0.7, 0.5% L-arabinose and 0.4 mM IPTG were added into the medium, and the culture was further incubated at 30 °C for 22 h. The genetically engineered bacteria were collected and eGVs were extracted by a previously published protocol. hGVs were biosynthesized and extracted from *Halobacterium NRC-1* according to our previous reports 30,31. Briefly, the GVs-containing bacterial culture was transferred into a separatory funnel. When the bacteria floated to the top, the lower liquid (medium and debris) was carefully discarded. Next, an equal volume of TMC lysis buffer was added. The bacteria were lysed for 24 h to release GVs. The lysis mixture was centrifuged at 300 g for 4 h at 4 °C. The lower liquid was removed and the GVs were washed with PBS for 3-4 times through centrifugation floatation method.

**Characterization of hGVs, eGVs and Sonovue:** The particle size and zeta potential of hGVs, eGVs and Sonovue were measured by a ZS XPLORER particle size analyzer. The morphology of hGVs and eGVs was examined using a GEM-F200 transmission electron microscope, while the morphology of Sonovue was observed with a Leica inverted microscope. The concentrations of hGVs and eGVs were determined by the optical density (OD) using a Nanodrop 2000c spectrophotometer.

**GC-MS detection of hGVs and eGVs:** An Agilent 7890A gas chromatograph with a dual FID+TCD detector was used. The temperature program started at 60 °C for 10 min, ramped to 180 °C at 20 °C/min, and held for 5 min. The TCD detector was set to 250 °C, using helium at 5 mL/min. CO_2_, N_2_, Xe, and H_2_S were analyzed via HP-Plot/Q column (2.8 to 7 min), and gases like hydrogen, oxygen and methane were separated with the HP-molesieve column. C1-C6 hydrocarbons were detected using an alumina column with an FID detector set to 300 °C. 1 mL sample was purged with helium, heated to 80 °C for 30 min, and analyzed by gas chromatography.

**In vitro imaging of hGVs, eGVs and Sonovue:** First, 2% agarose solution was prepared by dissolving 2 g of agarose in 100 mL of boiling deionized water, then poured into molds to form the well-like agarose phantom. The bubble concentrations of hGVs and eGVs were adjusted to OD_500_ values at 0.5, 1.0, 1.5, 2.0, 2.5, and 3.0. Two-fold diluted samples at OD_500_ = 2.5 and 5-fold diluted samples at OD_500_ = 3.0 were used for the in vitro imaging. Sonovue samples were prepared according to product instruction and further diluted to 100-, 20-, 10-, 5-, and 2-fold solutions. 100 µL of bubble samples at different concentrations were added into the agarose phantom. Imaging was performed with the ZS3 Ocular ultrasound machine, using a mechanical index (MI) of 0.12 and 1% contrast mode. Ultrasound signal intensities were analyzed using the software equipped in ultrasound imaging machine.

**Animal model:** Male SD rats (200-250 g) were purchased from Guangzhou Lingfu Topu Biological Co., LTD. All procedures were approved by the Animal Experiment Center of Xinjiang Medical University (Ethical approval number: IACAC-20220309-39). The rats were anesthetized, intubated, and connected to a ventilator and BL-420F system for stable respiration. A thoracotomy was performed at the 3rd and 4th intercostal spaces, and the left anterior descending artery was ligated with 7-0 sutures. Echocardiography was used to observe the successful establishment of MI model (Figure S12A), and ECG was used to monitor the elevated ST segment (Figure S12B). TTC staining was used to confirm the infarcted areas (Figure S12C).

**In vivo contrast imaging:** Rats were anesthetized with 5% oxygen and 1.5% isoflurane, placed supine on an imaging platform at 37 °C, and secured with surgical tape. Hair was removed from the area between the sternum and diaphragm using depilatory cream. The ultrasound probe was positioned and moved using a rail system, with B-mode imaging of the left parasternal area to obtain parasternal long-axis images. In contrast mode, imaging was performed at 13 MHz with a mechanical index of 0.12 using the L20-5 ultrasound system. 250 µL of hGVs (OD_500_ at 2.8, 3.0 and 3.2), eGVs (OD_500_ at 3.0) or Sonovue stock solution were injected via the tail veins (Figure S14).

**Immunofluorescence analysis of bubble penetration:** hGVs and eGVs were modified with Cy3-NHS and Sonovue was stained by Dil dye for the convenience of detection. Briefly, for the labeling of GVs, 500 µL of the Cy3-NHS solution (1 mg/mL) was added to the solution of hGVs and eGVs at OD 3.0 and incubated overnight on a shaker at 4 °C, followed by centrifugation at 300 g for 2 h to remove any free dyes. For the staining of Sonovue, 1 mg/mL Dil was mixed with 5 mL Sonovue, following washing as stated above. 250 µL fluorescence labeled GVs at OD 3.0 or Sonovue bubbles were systemically administrated via the tail vein into rats with myocardial infarction. After 5 min, the animals were euthanized and the hearts were removed to prepare frozen sections. The fluorescence signals were observed by a Leica inverted fluorescence microscope.

**Immunofluorescence analysis of inflammatory factor expression:** Tissue paraffin sections of infarcted myocardial tissues were deparaffinized, washed and treated in citrate buffer (pH 6.0) using microwave antigen retrieval (8 min medium power, 7 min low power). After that, sections were incubated with 3% hydrogen peroxide for 25 min to block endogenous peroxidase activity. The slides were washed three times with PBS (5 min each) and blocked with BSA for 30 min. The primary antibodies (IL-6 1:3000, TNF-α 1:3000, IL-1β 1:200) were applied, followed by incubation with HRP-labeled secondary antibodies for 50 min at room temperature. Nuclei were stained with DAPI for 10 min, and sections were sealed with anti-fluorescence mounting medium and observed by a Leica inverted fluorescence microscope.

**Transmission electron microscopy of tissues:** MI rats were intravenously injected with Sonovue, hGVs or eGVs, then sacrificed 30 min later. Tissue samples (2 mm × 2 mm) from the infarcted area were fixed in electron microscopy fixative at room temperature for 2 h, followed by fixation with 1% glutaraldehyde in 0.1 M PBS (pH 7.4) for 2 h in the dark. Samples were rinsed three times with PBS (15 min each), dehydrated and processed for infiltration and embedding. The blocks were polymerized at 60°C for 48 h. Ultrathin sections (60 - 80 nm) were cut and placed on 150-mesh copper grids, stained with 2% uranyl acetate in ethanol for 8 min, washed with 70% ethanol, and then stained with 2.6% lead citrate for 8 min. Grids were dried overnight and examined under a transmission electron microscope.

**HE and Masson trichrome analysis:** HE and Masson trichrome staining kits (Servicebio, China) were used. Briefly, for HE staining, samples were fixed in 4% paraformaldehyde and embedded in paraffin. Sections were dehydrated through xylene, absolute ethanol, and 75% ethanol, then washed with tap water. Hematoxylin staining was performed for 3-5 min, followed by differentiation, bluing, and dehydration through 85% and 95% alcohol. The sections were stained with eosin for 5 min, cleared in absolute ethanol and xylene, and mounted with neutral gum. For Masson staining, sections were incubated in Masson A solution at 37 °C for 30 min, then stained with a mixture of Masson B and C solutions for 3 min, followed by differentiation in 1% hydrochloric acid ethanol for 1 min. Sections were then stained in Masson D solution for 6 min, treated with Masson E solution for 2 min, and then stained in Masson F solution for 5 min. Sections were mounted with neutral gum. The HE- and Masson-stained sections were observed by a Leica inverted microscope.

**Oxygen delivery by hGVs combining with acoustic irradiation:** To assess the amount of oxygen released from hGVs, 1 mL hGVs solution at OD_500_ 2.0 was received with acoustic irradiation by the transducer equipped in the Vevo 2100 system (VisualSonics). The burst was used for 5 times with 3-seconds interval. The concentrations of oxygen in hGVs solution before and after burst was measured using a solution oxygen meter (S4-Field Kit) and recorded as the initial oxygen level. In the animal experiment, after MI rat model was established, hGVs (OD_500_ 3.0 ml/kg body weight) or 200 µL of Sonovue were injected via the tail vein at 12, 72, and 120 h post infarction. Ultrasound imaging was performed on the anterior wall of the left ventricle of hearts. Burst was applied to destruct these bubbles and to release gases from bubbles, with 5 s interval for a total 5 min duration.

**Left ventricular function analysis:** Cardiac ultrasound was performed on rats using the Vevo 2100 system (VisualSonics). For B-mode imaging, we used a 21 MHz MS250 linear array probe for optimal resolution and penetration. The M-Mode sweep speed was set at 1200 Hz for precise ventricular function measurements. M-mode echocardiography was used to calculate key cardiac indices, including left ventricular ejection fraction (LVEF), fractional shortening (LVFS), end-diastolic and end-systolic left ventricular internal diameters (LVID; d, LVID;s), interventricular septum thickness at end-diastole and end-systole (IVS; d, IVS;s), and left ventricular posterior wall thickness at end-diastole and end-systole (LVPW; d, LVPW;s).

**Biosafety:** Six healthy rats were divided into an experimental group and a control group. The rats in the experimental group were injected with 250 µL of hGVs (OD 3.0), while the control rats were intravenously injected with equal volume of saline. Tissue samples, including heart, liver, spleen, lung and kidney, were processed for paraffin embedding, sectioning, and HE staining to observe tissue damage. Blood samples were collected to assess liver and kidney function. The levels of alanine aminotransferase (ALT), aspartate aminotransferase (AST), urea (UREA), creatinine (CREA), and blood urea nitrogen (BUN) were measured according to the standard method.

**Statistical analysis:** Statistical analysis was performed using SPSS version 26. Data normality was assessed with the Shapiro-Wilk test. Normally distributed data are presented as mean ± standard deviation, while non-normally distributed data are reported as the median. One-way ANOVA was used for inter-group comparisons, with LSD tests conducted to evaluate specific differences between two groups. A p-value of less than 0.05 was considered statistically significant.

## Supplementary Material

Supplementary figures and video legends.

Supplementary video 1.

Supplementary video 2.

Supplementary video 3.

Supplementary video 4.

Supplementary video 5.

Supplementary video 6.

Supplementary video 7.
