# Supplementary material for: Biosynthetic gas vesicles as a novel ultrasound contrast agent for diagnosing and treating myocardial infarction
Source: Theranostics. 2025 Jul 28;15(16):8553–68. doi: 10.7150/thno.118543 (PMC12374649; doi:10.7150/thno.118543)
Supplement: Supplementary file 1 — Supplementary figures and video legends. [file thnov15p8553s1.pdf]

Biosynthetic gas vesicles as a novel ultrasound contrast agent for diagnosing and treating myocardial infarction

Zihang Wang[1], Maierhaba·Yibulayin[1], Kezhi Yu, Tingting Liu, Lina Guan, Baihetiya Tayier, Lingjie Yang, Shangke Chen, Yuming Mu*, Fei Yan*

Z. Wang, M. Yibulayin, L Guan, B. Tayier, L.Yang, Dr Y. Mu
Department of Echocardiography, The First Affiliated Hospital of Xinjiang Medical University, Xinjiang Key Laboratory of Ultrasound Medicine, Xinjiang 830000, China. E-mail: mym1234@126.com
K.Yu
Department of Ultrasound, Fifth Affiliated Hospital of Sun Yat-sen University, Zhuhai, 519000, Guangdong, China
T. Liu
Department of Ultrasound, The Second People's Hospital of Shenzhen, The First Affiliated Hospital of Shenzhen University, Shenzhen, P. R. China
S. Chen
Xinjiang Key Laboratory of Ultrasound Medicine. No. 137, Liyushan South Road, Xinjiang 830000, China
Z. Wang, Prof. F. Yan
Key Laboratory of Quantitative Synthetic Biology, Shenzhen Institute of Synthetic Biology, Shenzhen Institutes of Advanced Technology, Chinese Academy of Sciences, Shenzhen 518055, China
E-mail: fei.yan@siat.ac.cn;
[1]Zihang Wang and Maierhaba·Yibulayin contributed equally to this work.
*These authors are co-corresponding authors.

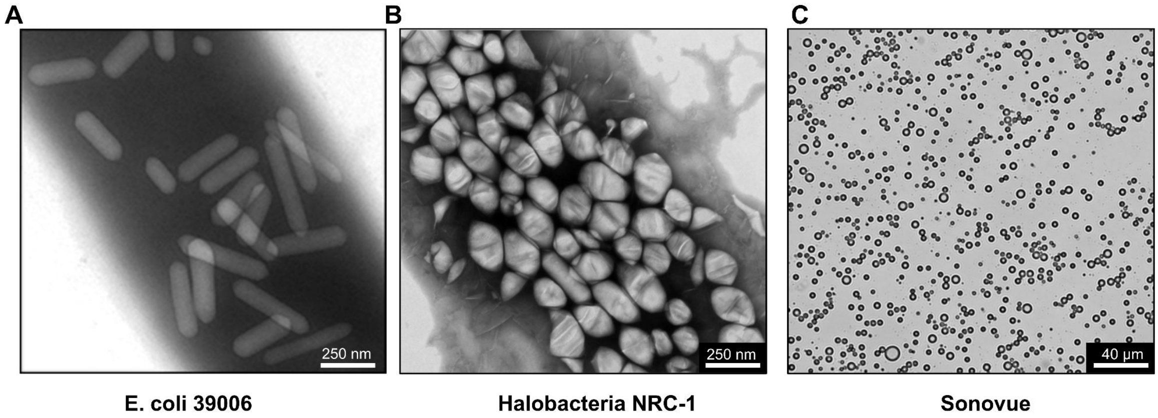

Figure S1. The shape of GVs and Sonovue. **(**A) Image of eGVS in the genetically engineered *E. Coli*BL21(A1) transformed with plasmides harboring GVs-encoding gene cluster from *Serration. 39006*. (B) Image of hGVs in the *Halobacteria NRC-1*. (C) Image of Sonovue microbubles under microscope.

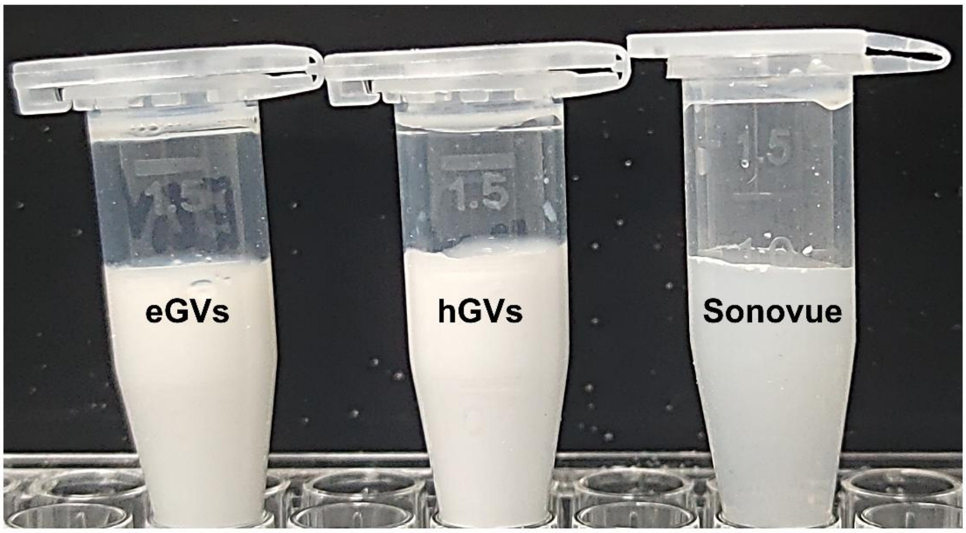

Figure S2. The images of GVs and Sonovue. eGVs and hGVs solutions at the concentration of OD $_{500}$ 3.0. The original concentration of Sonovue through adding 5mL saline into the dry powder.

Test result

number	Test	Result	Percent	Unit	Method
1	CO_2	8.65895	2.41%	mg/L	GC-MS
2	O_2	97.88530	27.30%	mg/L	GC-MS
3	N_2	252.05899	70.29%	mg/L	GC-MS
hGVs					

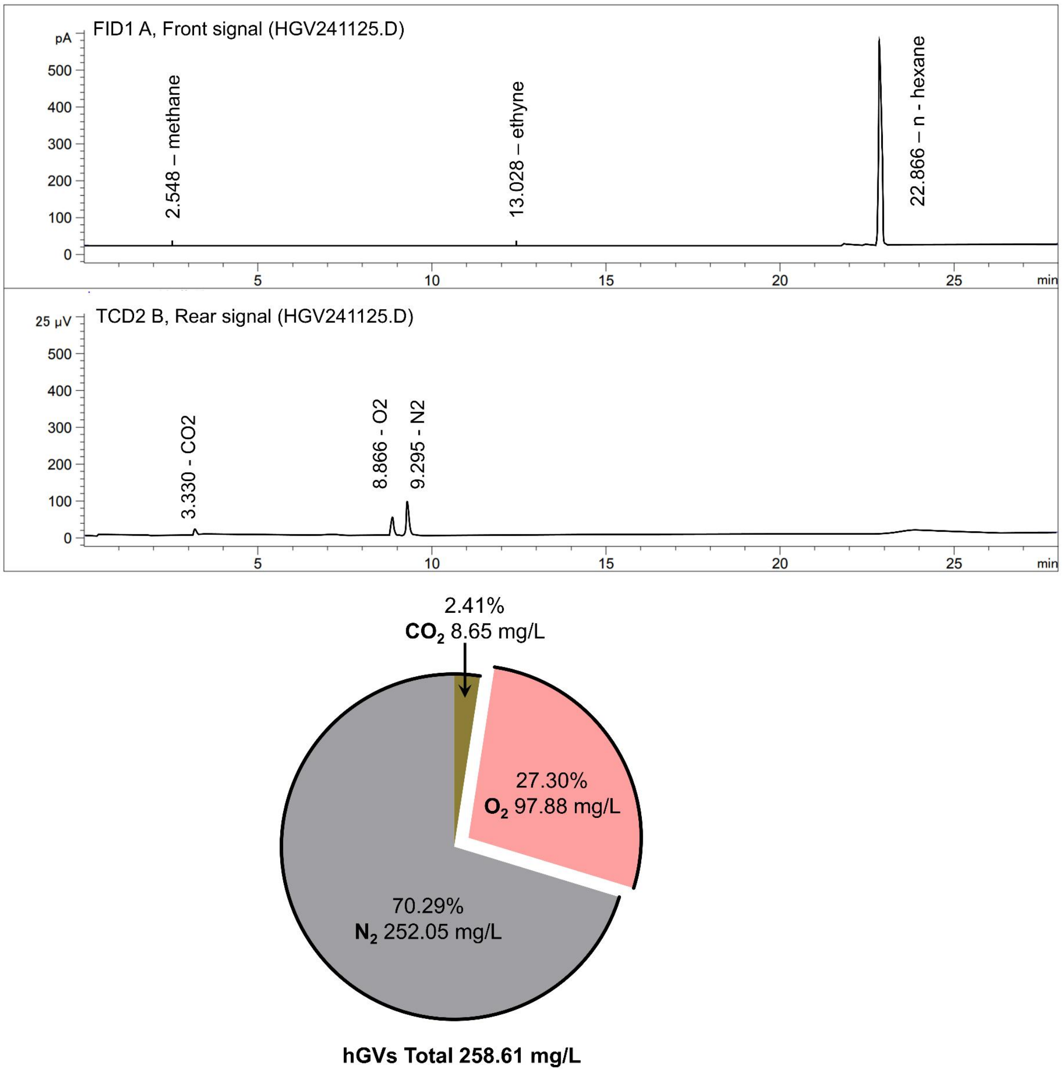

Figure S3. The composition and content of gases in hGVs. When the concentration hGVs is at OD_{500} 2.0. There are 8.65 mg/L CO_2, 97.90 mg/L O_2, N_2 content was 252.06 mg/L, accounting 2.42%, 27.30% and 70.29% volume ratio, respectively.

Test result

number	Test	Result	Percent	Unit	Method
1	CO_2	6.36903	3.35%	mg/L	GC-MS
2	O_2	48.72250	25.59%	mg/L	GC-MS
3	N_2	135.29122	71.06%	mg/L	GC-MS
eGVs					

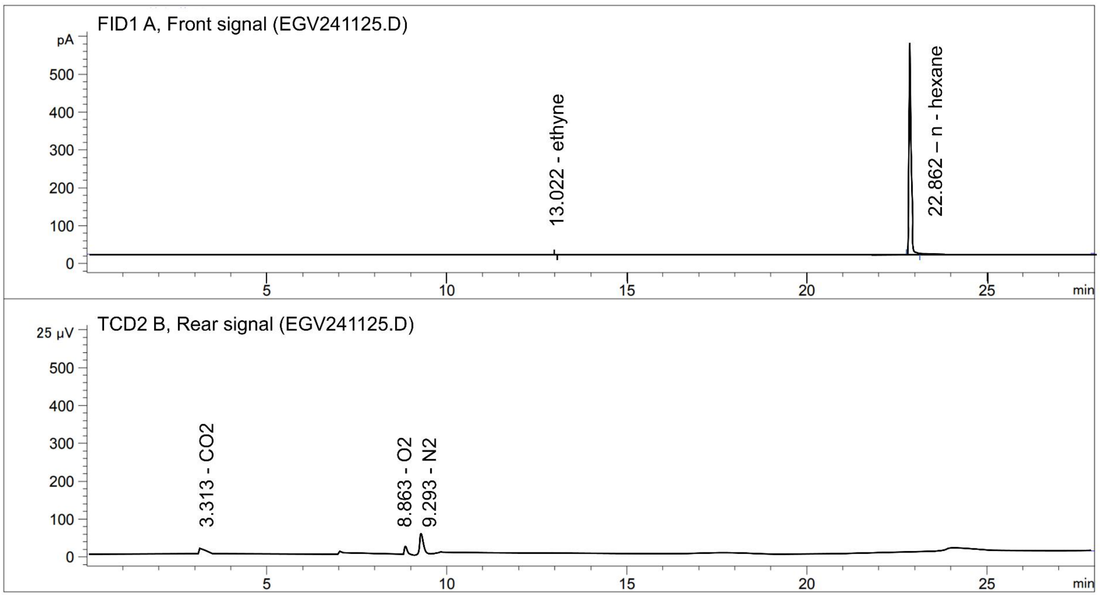

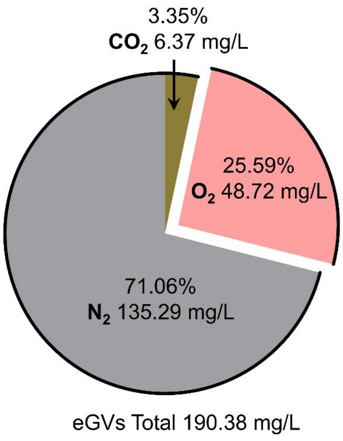

Figure S4. The composition and content of gases in eGVs. When the concentration of hGVs is at OD_{500} 2.0. There are 6.36 mg/L CO_2, 48.72 mg/L O_2, content was 135.29 mg/L, accounting 3.34%, 25.59% and 71.06% volume ratio, respectively.

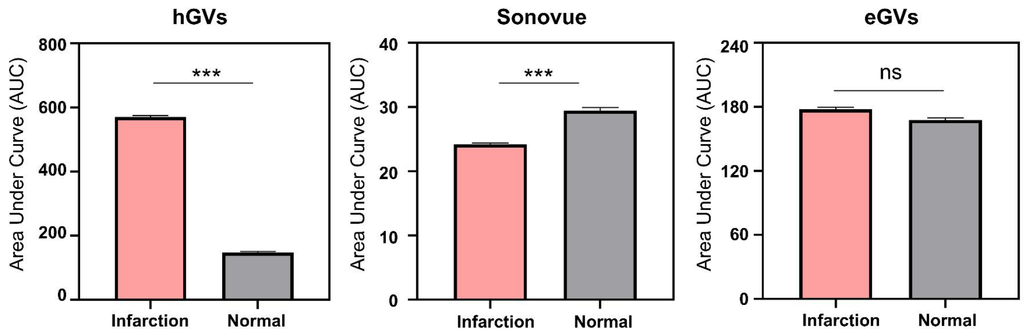

Figure S5. Quantification of the AUC for the infarcted and non-infarcted regions of MI rats received with hGVs from (Fig. 4B, 4C and 4D). (n = 3).

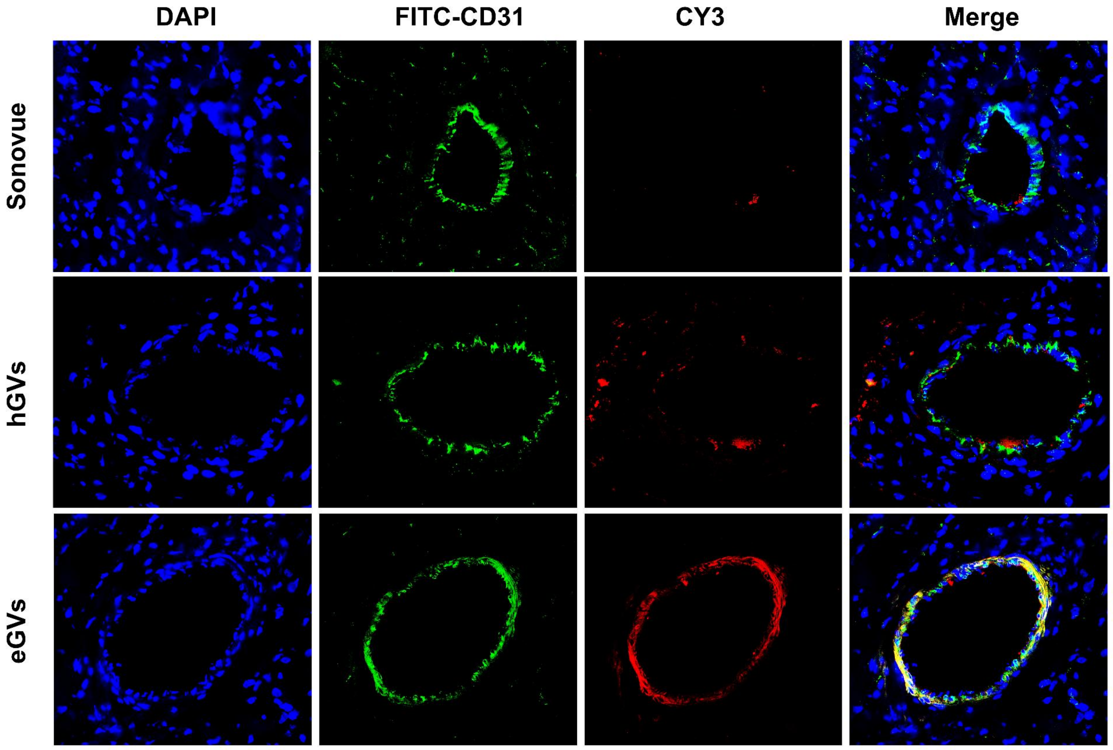

Figure S6. Immunofluorescence staining of the myocardial tissue in the normal area after systemic administration of fluorescence-labeled bubbles. Revealing only a small number of hGVs penetrated into the surrounding myocardial tissue through the vascular wall. The eGVs primarily accumulated around endothelial cells while Sonovue was predominantly confined to the blood vessels. Green fluorescence indicates CD31 positive cells, blue fluorescence indicates DAPI stained nuclei, and red fluorescence represents fluorescence-labeled bubbles.

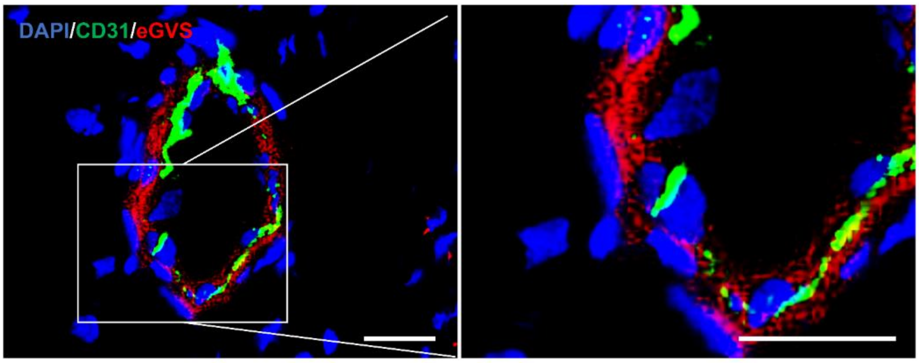

Figure S7. Immunofluorescence staining of microvessel endothelial cells in the left anterior wall of the MI group; green fluorescence indicates CD31 positivive cells, blue fluorescence indicates DAPI stained nuclei, and red fluorescence represents eGVs. (scale bar: 20 μm).

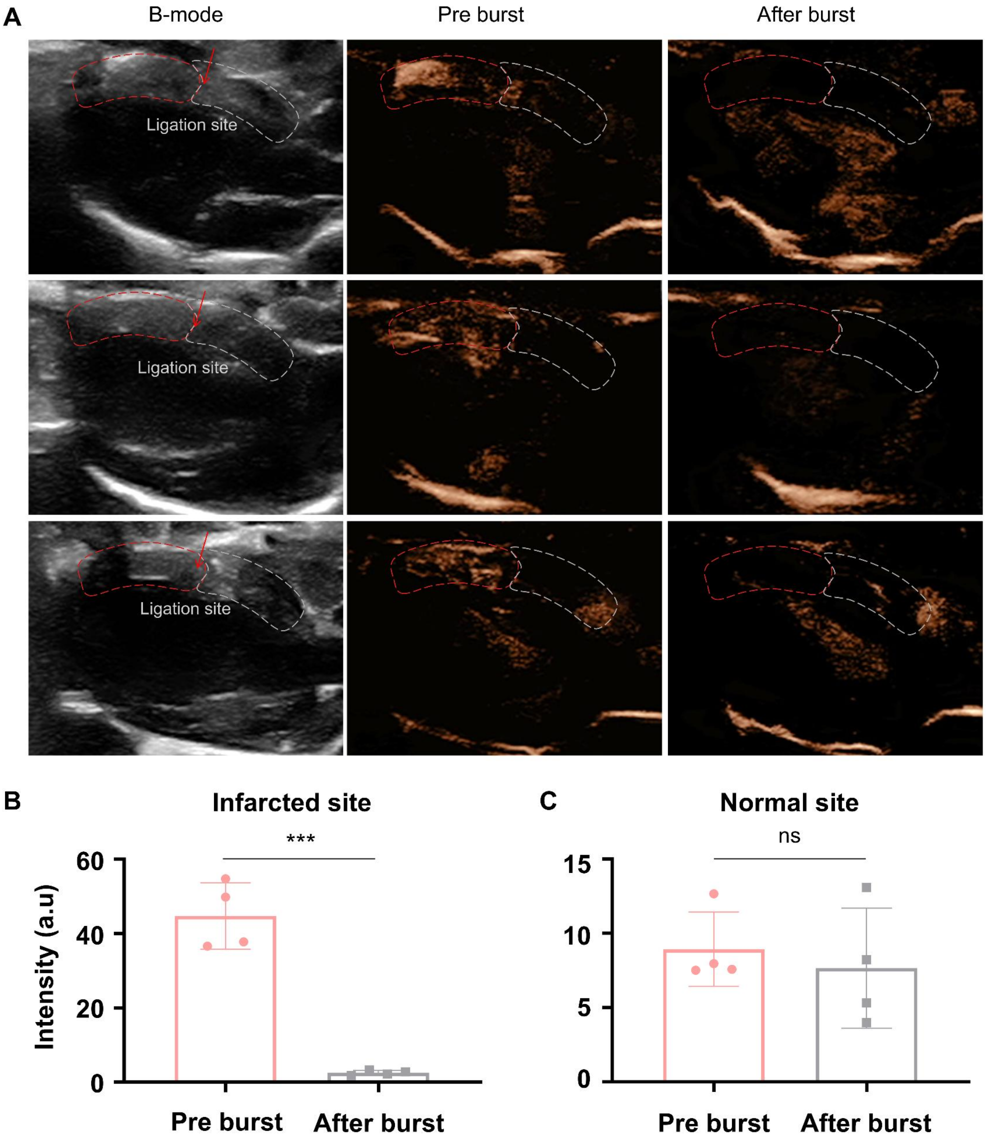

Figure S8. Ultrasound contrast imaging of eGVs, hGVs, Sonovue in the MI rat hearts. (A) the contrast signals of myocardial infarction site and normal site in the anterior wall of left ventricle were observed after 20 min post injection of hGVs in rats with myocardial infarction. (B) Quantification of left ventricular infarct contrast signals before and after ultrasonic burst. (C). Quantitative contrast signal of normal myocardium in left ventricle before and after ultrasonic burst. (n = 4).

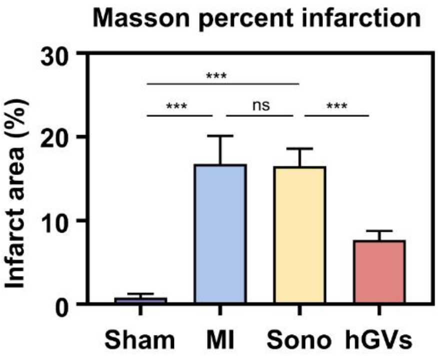

Figure S9. Quantitative evaluation of fibrotic area by Masson staining after treatment of MI rats with hGVs or Sonovue.

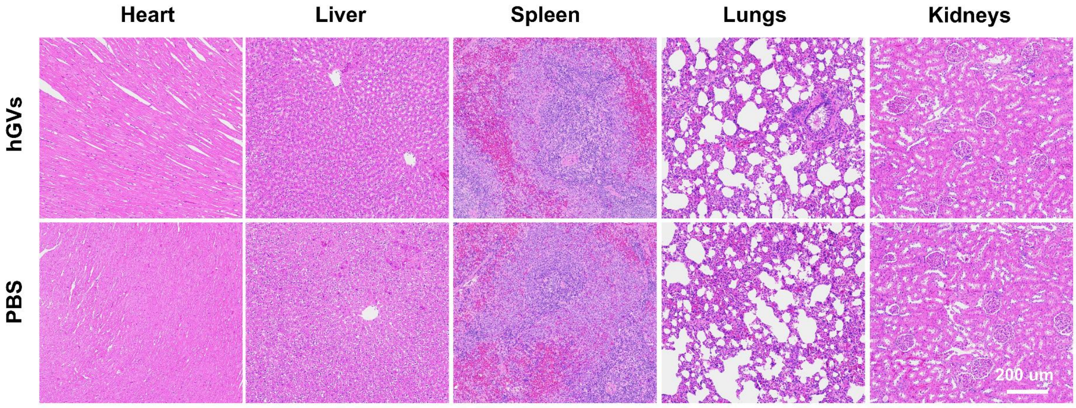

Figure S10. HE staining histological evaluation of heart, liver, spleen, lung and kidney in hGVs group and PBS-treated control group (n = 3).

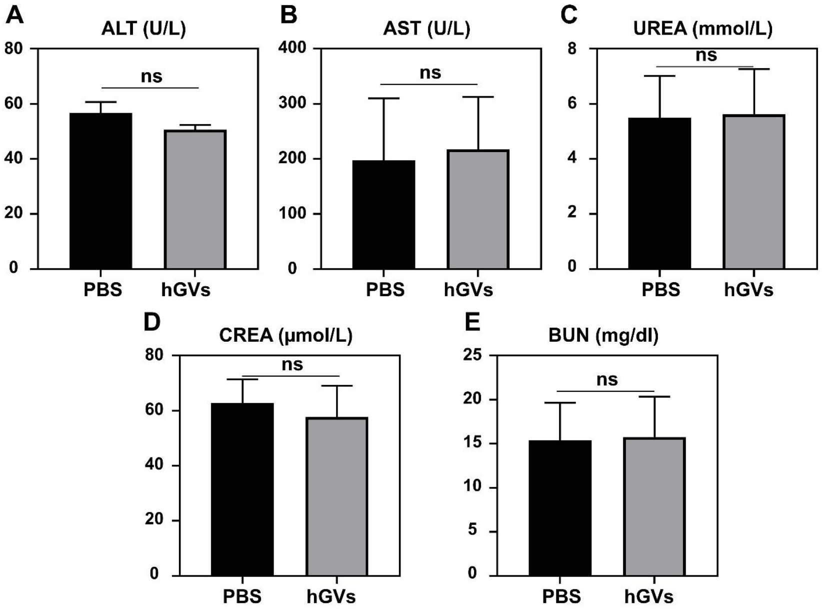

Figure S11. (A-E). Detection of levels of ALT (alanine aminotransferase), AST (aspartate aminotransferase), urea (UREA), creatinine (CREA), and blood urea nitrogen (BUN) after injection of PBS or hGVs (n = 3).

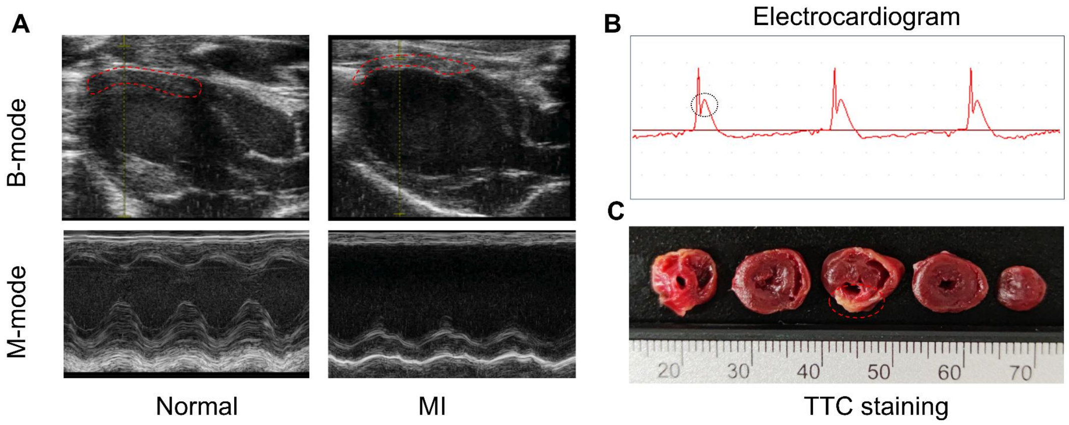

Figure S12. (A) Left ventricular long-axis view of normal rats and myocardial infarction rats in the B mode and M mode. (B) Electrocardiogram after modeling myocardial infarction. (C) TTC staining after myocardial infarction.

Primers	Sequence(5'to3')
GvpA-F	AAGAAGGAGATATACATATGATGGCTAAAGTACAAAAATCAACAG
GvpA3-R	GTTTCTTCTATGACGGACGGT
GvpA3-F	ACCGTCCGTCATAGAAGAAAC
GvpC-R	TGATGATGATGGTGCATATGCTACTCGCTCGGCTGTGATA

Figure S13. Primers used in this study.

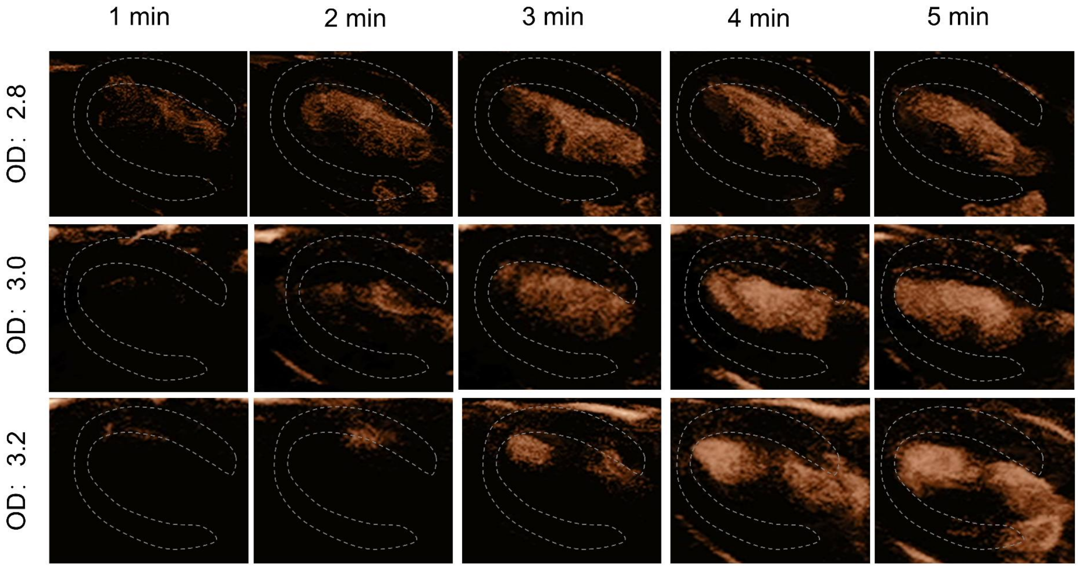

Figure S14. The cardiac contrast image of rat hearts with hGVs at OD_{500} = 2.8, 3.0, or 3.2.

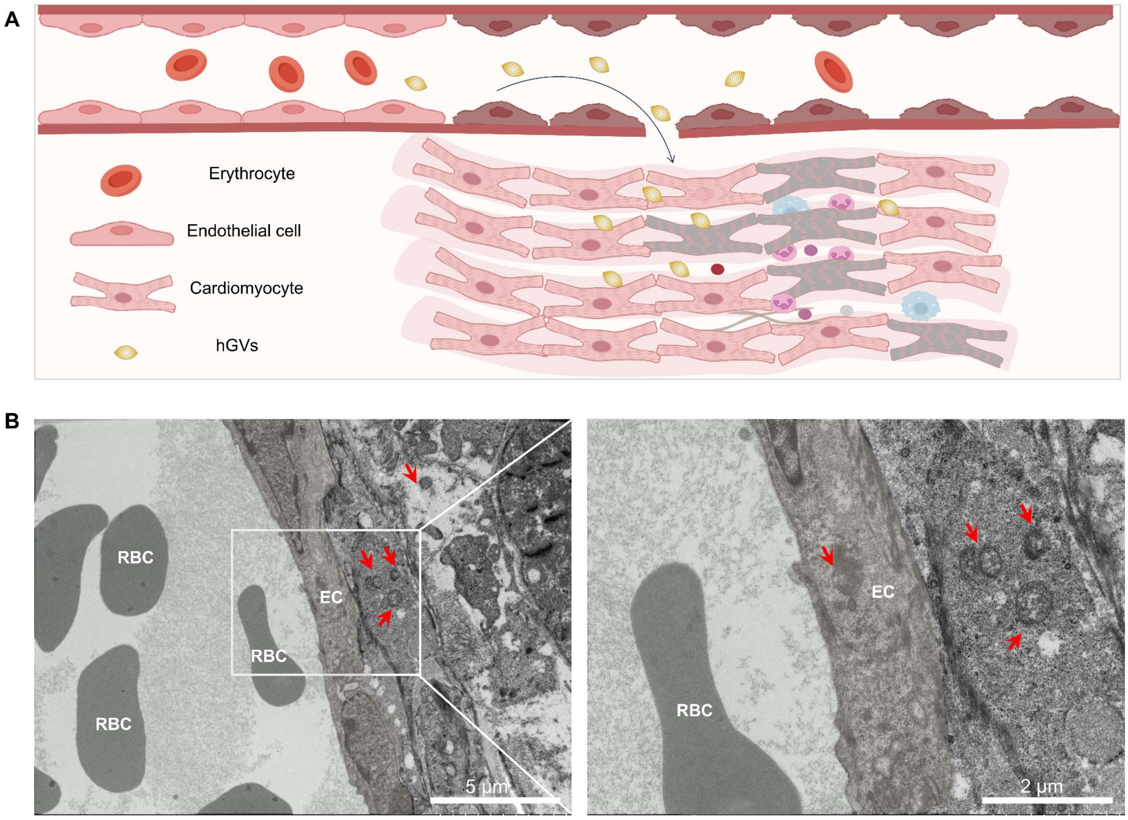

Figure S15. (A) Diagram of hGVs crossing blood vessels into surrounding tissues. (B) hGVs could be observed at the site of the damaged myocardial infarction.

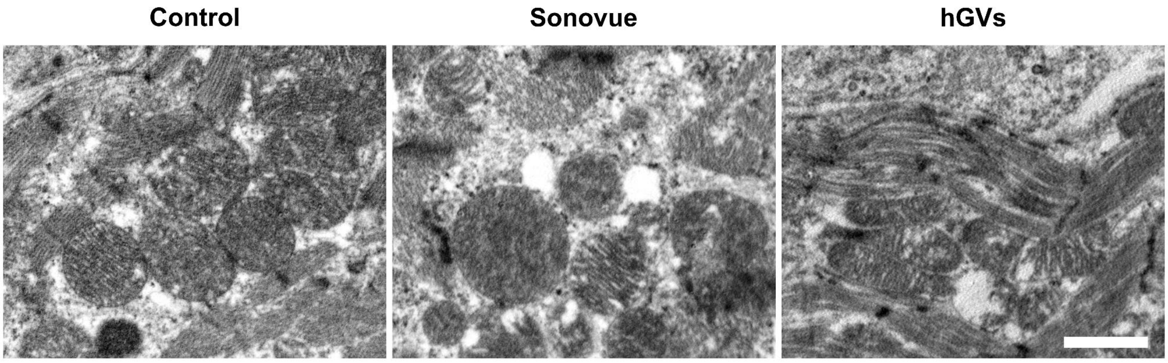

Figure S16. TEM images of cardiac myocytes from different treatment groups. Control group showing mitochondria with blurred cristae structures and signs of swelling under hypoxic conditions. Sonovue group with similar mitochondrial changes as the control group. hGVs group demonstrating preserved mitochondrial structure with clear and well-defined cristae. Scale bar: 1 μm.

Video 1. hGVs imaging in rats with myocardial infarction (Video play speed ×24).

Video 2. hGVs imaging in SHAM group rats (Video play speed ×24).

Video 3. eGVs imaging in rats with myocardial infarction (Video play speed ×12).

Video 4. eGVs imaging in SHAM group rats (Video play speed ×12).

Video 5. Sonovue imaging in rats with myocardial infarction (Video play speed ×24).

Video 6. Sonovue imaging in SHAM group rats (Video play speed ×24).

Video7. 20 min after hGVs injection, images before and after ultrasonic blasting were performed.
